# Temporal response of post-activation performance enhancement induced by a plyometric conditioning activity

**DOI:** 10.3389/fspor.2023.1209960

**Published:** 2023-06-27

**Authors:** Marcos Vinicius Casais Barreto, Juliana Ferreira da Silva Telles, Marcela Rodrigues de Castro, Thiago Teixeira Mendes, Caio Portela Rodrigues, Victor Hugo de Freitas

**Affiliations:** ^1^ Postgraduate Program in Rehabilitation Sciences, Institute of Health Sciences, Federal University of Bahia, Salvador, BA, Brazil; ^2^ Department of Physic Education, Faculty of Education, Federal University of Bahia, Salvador, BA, Brazil

**Keywords:** power, countermovement jump, exercise, post-activation potentiation, plyometrics

## Abstract

**Introduction:**

To better understand the post-activation performance enhancement (PAPE) effect promoted by a plyometric conditioning activity (CA), the aim of this study was to investigate the temporal response of PAPE after a plyometric CA.

**Methods:**

Fourteen healthy and active adults visited the laboratory 3 times, with an interval of 7 days between each visit. On the first day they were familiarized with the countermovement jump (CMJ) test and plyometric CA. In the second and third visits, participants performed either plyometric CA or control (remaining seated) in a crossover design. The CMJ test was performed pre and 1-, 3-, 6-, and 9-min post the plyometric CA or control. The comparisons were performed using the repeated measure two-factor ANOVA and Bonferroni adjustment (significance level adopted *P* ≤ 0.05).

**Results:**

Time (*P* < 0.01), condition (*P* < 0.01), and interaction (*P* < 0.01) effects were reported for CMJ comparisons. For the control condition, CMJ increased at 3 min compared to pre (*P* = 0.03) and at 3 min compared to 1 min (*P* = 0.03). For the plyometric CA, CMJ increased at 1- (*P* < 0.01), 3- (*P* < 0.01), and 6-min (*P* = 0.02) compared to pre. For condition comparisons, CMJ was different at 1- (*P* < 0.01), 3- (*P* < 0.01), 6- (*P* < 0.01), and 9-min (*P* = 0.02). The Effect size of the comparisons of all moments compared to pre was null (d < 0.20) for control and small (d < 0.50) for plyometric CA.

**Discussion:**

It is possible to conclude that the plyometric CA promoted a PAPE effect for up to 9-min. Strength and conditioning coaches and practitioners may consider multiple sets of plyometric CA to produce immediate enhancement of power in the lower limbs.

## 1. Introduction

Post-activation performance enhancement (PAPE) is an acute improvement in voluntary muscular performance (strength production) as a result of a previous voluntary conditioning activity (CA) (1, 2). PAPE is one of the main objectives of a warm-up, and its phenomenon may be explained by phosphorylation of myosin regulatory light chains (at least in the earliest stages), fluid shifts into the working muscles, and increased muscle activation (2). Traditional high- or moderate-intensity strength exercises are frequently used to induce PAPE (3). Furthermore, PAPE has been reported post plyometric exercises (3), which involve the stretch-shortening cycle to store energy and produce more powerful movement (4, 5). Plyometric exercises use different types of jumps (bilateral, unilateral, bounds, hops, and drop jumps) (4, 5) and do not depend on equipment and implements, demonstrating a practical advantage compared to traditional high- or moderate-intensity CA.

It was suggested that the rest period after CA influences the magnitude of PAPE (3, 6). Apparently, fatigue and potentiation coexist after a CA (7), and PAPE is reported only if the potentiation is greater than the fatigue (3). Therefore, based on two meta-analyses (3, 6), Bullosa (1) suggested that greater PAPE is found 5–10 min after the CA. However, PAPE may be inﬂuenced by the type of CA, and improvement in voluntary muscular performance may be reported 0.3–4 min after a plyometric CA (3). For example, improvement in countermovement jump (CMJ) performance was reported 1–5 min after multiple sets of plyometric CA performed by professional rugby union players (8). However, the reported study investigated the PAPE effect up to 5 min after the plyometric CA and no effect >5 min was shown (8). In male collegiate soccer players, for example, an improvement in CMJ was reported 10 min after a plyometric CA (9). Another study investigated the PAPE effect 7 and 15 min after top-level sprinters executed drop-jumps and did not find positive effects (10). The inconsistency of the results reported (8–10) and the limited number of studies investigating the PAPE effect after a plyometric CA (3) contribute to the difficulty in understanding the temporal response of PAPE after this CA, making new investigations necessary.

To better understand the PAPE effect promoted by a plyometric CA, the aim of this study was to investigate the temporal response (up to 9 min) of PAPE after a plyometric CA. This investigation will help coaches and practitioners to manage the time of rest between the plyometric CA and subsequent exercise. The hypothesis raised was that a plyometric CA could promote PAPE and an effect would be shown up to 9 min after CA.

## 2. Materials and methods

This is a randomized controlled clinical trial with a crossover design. To verify the temporal response of PAPE induced by a plyometric CA, participants performed a CMJ test pre and 1-, 3-, 6-, and 9 min post multiple sets of plyometric exercises (plyometric CA) or control, performed seven days apart. The CMJ was selected as the performance test because it is a simple, practical, reliable, and validated field test to estimate power of the lower limbs (11) and a sensitive test to monitor neuromuscular status (12). Furthermore, the jump height performed is associated with the ability to generate yank (the first time derivative of force) (13).

### 2.1. Subjects

In total, 14 healthy active adults (male = 11; female = 3) participated in the study. The inclusion criteria were: aged between 18 and 45 years; absence of illness or musculoskeletal limitations; self-reported practice of at least 150 min of moderate physical activity per week; and absence of medicines or substances that could interfere in the study. In addition, subjects were instructed to abstain from exercise, not to consume alcoholic drinks, to maintain their habitual meals before and on the day before the data collection, and not to consume caffeine for at least 12 h prior to the data collection. The exclusion criteria adopted were: occurrence of musculoskeletal injuries and missing collection days. No participants were excluded. The participants received a detailed explanation about the purpose of the study and about the experimental procedures before signing a consent form giving their free and voluntary agreement to participate in the study. The study was approved by the Human Research Ethics Committee (CAAE: 51101021.0.0000.5531) and followed the principles established in the World Medical Association Declaration of Helsinki.

### 2.2. Procedures

#### 2.2.1. Design

**Figure 1:**
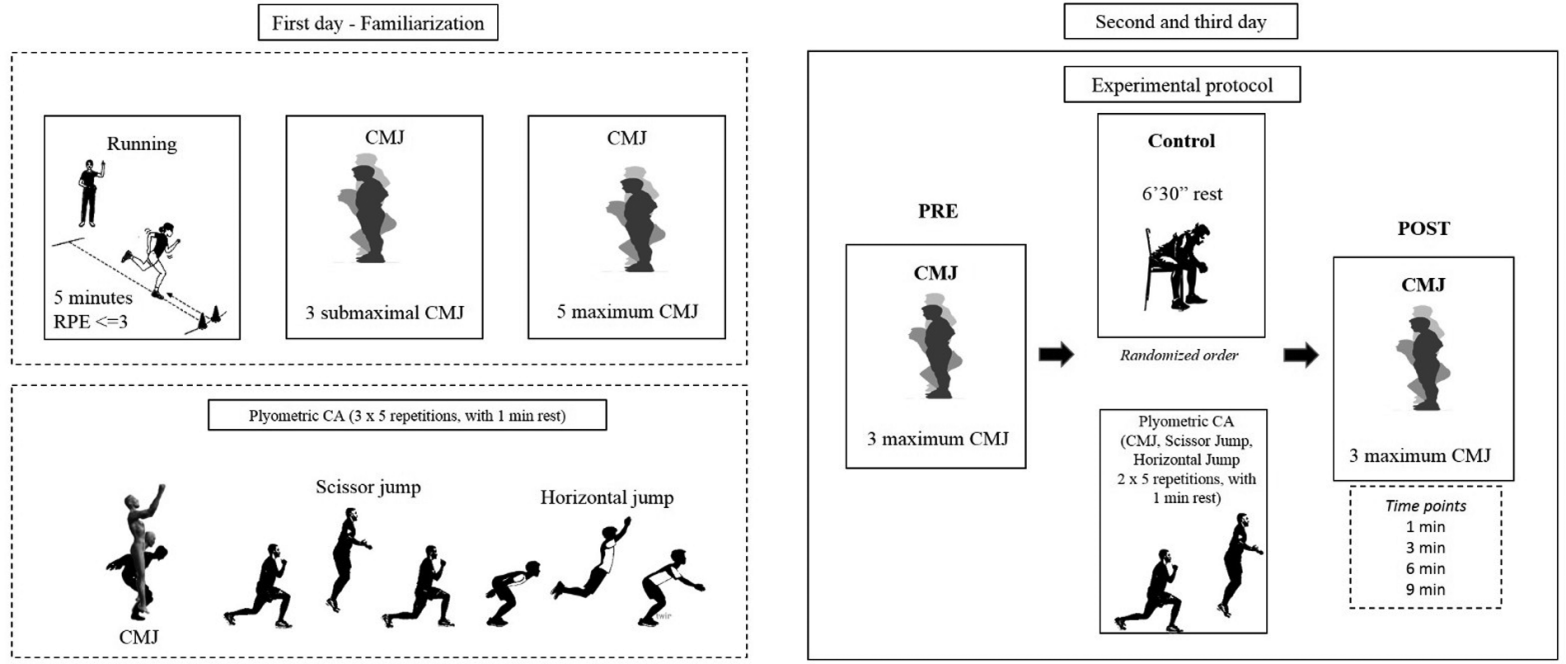
Study design. Plyometric conditioning activity, RPE = rating of perceived exertion, CMJ = countermovement jump.

The participants visited the laboratory 3 times, 7 days apart, at the same time of the day (8 and 10 a.m.). On the first day, the inclusion criteria were checked, and the participants signed the consent form. They were then characterized through the variables age, weight, and height, and performed the familiarization with the test and plyometric CA. The familiarization consisted of a warm-up with 5 min running/walking with a perceived exertion ∼3 (Scale 0–10) (14), followed by 5 min of rest, 3 submaximal CMJ, and 5 maximal CMJ. The 5 maximal CMJs were used to analyze the reliability of the test. Subsequently, the plyometric CA (3 × 5 rep with 1 min of rest of CMJ, scissor jump, and horizontal jump = totalizing 45 jumps) was performed. In the second and third visits the participants performed the plyometric CA or the control in a cross-over design. Therefore, on the second day, 7 participants performed the plyometric CA and the other 7 performed the control, with inversion of procedures performed on the third day. The order of procedures was randomized using a code and an excel spreadsheet. The plyometric CA consisted of 2 × 5 rep of CMJ, scissor jump (2 × 5 rep for each leg), and horizontal jump with a 1 min rest between sets and exercises (totalizing 30 jumps). The control condition consisted of participants remaining seated on a chair for 6 min and 30 s (close to time spent performing the plyometric CA). The CMJ test was performed before (pre) and 1-, 3-, 6-, and 9 min post the plyometric CA or control. After both conditions and between the CMJ tests at post (1-, 3-, 6-, and 9 min), participants remained at rest in an orthostatic position. All procedures are shown in Figure 1.

#### 2.2.2. Countermovement jump test

The CMJ test was performed using the optical device New Fit Jump System (Cefise ®, São Paulo, BR), which was developed to measure ground contact time and flight time in vertical jumps. For the CMJ test, the participant started from the orthostatic position, performed a squat with a stretch-shortening cycle and immediately performed a jump. The participants were instructed to perform a maximum vertical jump and received feedback on each jump performed. Furthermore, they received motivational guidance through the following verbal stimulation: you can jump high (or you can jump higher), concentrate, prepare, go. The motivational guidance was standardized to reduce the chance of bias (15). Three CMJs were performed with arms akimbo, respecting a 15 s interval between them, during which the participants remained at rest in an orthostatic position. The average jump height of the 3 jumps was determined as the performance variable. The five CMJs performed during the familiarization were used for the reliability analysis *via* intraclass coefficient correlation calculation (single measure ICC = 0.95 [CI95% = 0.90–0.98], average measure ICC = 0.99 [CI95% = 0.98–1.00].

### 2.3. Statistical analysis

No outliers were identified using the *z*-score (z = (sample-mean)/standard deviation; outliers = z > 3). The data normality was confirmed by the Shapiro-Wilk test. A repeated measure ANOVA with two-factor (time and condition) was performed for between, within, and interaction comparisons. The multiple comparisons were performed using the Bonferroni adjustment. Sphericity was verified by Mauchly's test. Sphericity was not assumed for the time, and was interpreted by the Greenhouse-Geisser correction. These analyses were performed using SPSS (IBM® SPSS® Statistics 26.0). The significance level adopted was *P* ≤ 0.05. The effect size (ES: Cohen's d = (mean 2—mean 1)/standard deviation 1) of each moment compared to pre for control and plyometric CA was measured. The ES magnitudes were interpreted as follows: d (0.20) = small, d (0.50) = medium, d (0.80) = large (16).

A posteriori power (1—*β*) of CMJ comparisons was calculated using G*power (version 3.1.9.7, Franz Faul, University Kiel, Germany), including the partial eta square (ղ_p_^2^) values to calculate the effect size f, and considering an alpha error = 0.05, total sample size = 14, number of groups = 2, number of measurements = 5, correlation between repeated measurements = 0.5, and non-sphericity correction of 1.

## 3. Results

A description of the age, weight, height, and body mass index (BMI) of the participants is reported in Table 1.

**Table 1:** Description of age, weight, height, and body mass index of the participants.

	Mean ± SD
Age (years)	28.07 ± 7.63
Body mass (kg)	72.86 ± 12.12
Height (cm)	171.93 ± 5.88
Body mass index (kg/m²)	24.67 ± 4.15

The CMJ results at different moments from when control or plyometric CA were performed, and the mean difference between each moment and pre values are described in Table 2.

**Table 2:** Description of countermovement jump results at different moments when control and plyometric conditioning activity were performed.

Moments	Interventions	Mean ± SD (cm)	Mean difference (cm)	ES (Cohen's d)	ES magnitudes
Pre	Control	33.59 ± 8.70			
	Plyometric CA	33.71 ± 8.69			
1 min	Control	33.76 ± 9.19	0.17	0.02	Null
	Plyometric CA	37.30 ± 9.47	3.59	0.41	Small
3 min	Control	34.59 ± 9.24	1.00	0.11	Null
	Plyometric CA	36.45 ± 9.40	2.74	0.32	Small
6 min	Control	34.52 ± 9.28	0.93	0.11	Null
	Plyometric CA	36.04 ± 9.21	2.33	0.27	Small
9 min	Control	34.02 ± 8.72	0.43	0.05	Null
	Plyometric CA	35.58 ± 9.48	1.87	0.22	Small

Mean difference: comparisons of mean differences of each moment compared to pre for control and plyometric conditioning activity (CA). ES: effect size (Cohen's d) of each moment compared to pre for control and plyometric conditioning activity (CA). ES magnitudes: d (0.20) = small, d (0.50) = medium, d (0.80) = large.

**Figure 2:**
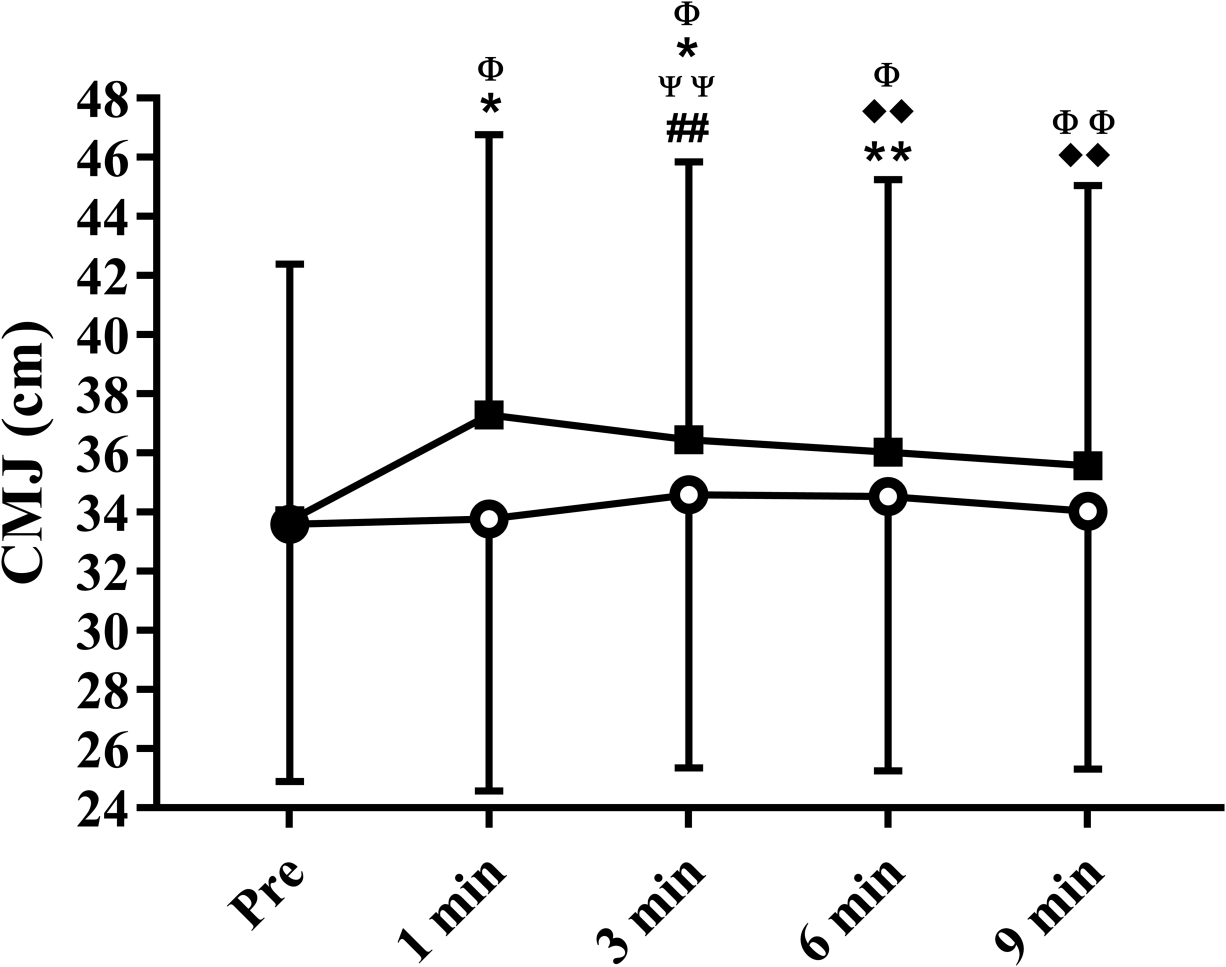
Mean ± standard deviation of countermovement jump results at pre and up to 9 min post control (white circles) and plyometric conditioning activity (black squares) interventions. #Difference from pre-value in control (##*P* < 0.05); *difference from pre-value in plyometric (*P < 0.01; **P < 0.05); Ψ difference from 1 min in control (ΨΨ P < 0.05). ♦ difference from 1 min in plyometric (♦♦ *P* Description of of age, weight, height, and body mass 0.05). Φ difference between conditions (Φ P < 0.01; ΦΦ P < 0.05).

Time (F = 11.91; ղ_p_^2^ = 0.48; *P* < 0.01), condition (F = 17.79; ղ_p_^2^ = 0.58; *P* < 0.01), and interaction (F = 12.61; ղ_p_^2^ = 0.49; *P* < 0.01) effects were found for CMJ comparisons. When control was performed, the CMJ increased at 3 min compared with pre (*P* = 0.03). Furthermore, the CMJ was higher at 3 min compared to 1 min (*P* = 0.03). No other time differences were reported for the control condition. When plyometric CA was performed, the CMJ increased at 1 min (*P* < 0.01), 3 min (*P* < 0.01), and 6 min (*P* = 0.02) but was not different at 9 min (*P* = 0.11) compared to pre. Furthermore, the CMJ was higher at 1 min compared to 6 min (*P* = 0.02) and 9 min (*P* = 0.02). No other time differences were reported for plyometric CA. For the condition comparisons, CMJ were not different at pre (*P* = 0.80), but was different at 1 min (*P* < 0.01), 3 min (*P* < 0.01), 6 min (*P* < 0.01), and 9 min (*P* = 0.02) (Figure 2). The ES (Cohen's d) and ES magnitudes are reported in Table 2. The ES magnitude of the comparisons of all moments compared to pre was null (d < 0.20) for control and small (d < 0.50) for plyometric CA.

The power values (1—*β*) of CMJ comparisons were 1.00 for time (f = 0.96), 1.00 for condition (f = 1.18), and 1.00 for interaction (f = 0.98).

## 4. Discussion

The main finding of the present study was that CMJ increased post the plyometric CA. The difference between the conditions at 1–9 min post interventions, in addition to the small ES reported for comparisons between post (1–9 min) and pre moments for plyometric CA led us to accept the hypothesis raised, showing that plyometric CA promoted PAPE effects up to 9-min after the CA.

In the present study, multiple sets of a plyometric CA (2 × 5 repetitions of 3 exercises, totalizing 30 jumps) increased the CMJ performance at 1-, 3-, and 6 min post CA. The use of multiple sets of a CA has been suggested previously (6), since it induces a larger PAPE effect than a single set of CA (3, 6). The result found is in accordance with results reported by Tobin and Delahunt (8), showing a PAPE effect 1-, 3-, and 5 min after multiple sets of a plyometric CA. The time response of PAPE after the plyometric CA reported was different to traditional high- and moderate-intensity CA, for which a PAPE effect was reported for >5 min of CA (1, 3, 6). A previous study with professional rugby players, for example, indicated increased performance in CMJ at 8- and 12 min post a preload stimulus of 3RM (repetition maximum) in the squat (17). One explanation for this divergent response was reported previously by Seitz and Haff (3), who suggested that a plyometric CA may produce less fatigue than a traditional high- or moderate-intensity CA, making it possible to observe an earlier PAPE effect. This suggestion is in accordance with the results shown by Sharma et al., (9), who reported a larger decrease in CMJ height 1 min post a heavy-resistance exercise than post a plyometric exercise. Although no mechanisms were analyzed to help explain the results found, the present study corroborates with the suggestion made by Seitz and Haff (3) that the PAPE effect may be reported earlier after a plyometric CA.

Another important result found was the higher values of mean difference between CMJ height 1 min after plyometric CA and pre compared to mean differences between other moments (3-, 6-, 9 min) and pre. Although no significant differences (based on *P* value) were reported for comparisons between mean differences, the higher values of CMJ at 1 min after the plyometric CA reinforce the idea that the PAPE effect may be reported earlier after a plyometric CA (3). Higher values of CMJ at 1 min (compared to 3- and 5 min) were found by Tobin and Delahunt (8), suggesting that plyometric CA produces an immediate enhancement in CMJ performance. The preferential recruitment of type II motor units during plyometric exercises (3) and the increase in the compliant muscle-tendon unit (8, 18) are other mechanisms than the net balance between fatigue and potentiation speculated to explain the earlier PAPE effect promoted by a plyometric CA. This speculation should be confirmed in future studies.

In accordance with frequent reports in the literature, that a greater PAPE is found >5 min after the CA (1, 6), it was hypothesized that plyometric CA could increase CMJ performance for up to 9 min after this CA. However, based only on the *P*-value reported for the between moment comparisons, the results found do not allow us to accept this hypothesis. This is in line with a previous study reporting that a PAPE effect was not shown 7 and 15-min after drop-jumps performed by top-level sprinters (10). On the other hand, it is important to highlight the 1,87 cm mean difference between CMJ height 9 min after the plyometric CA compared to pre. PAPE effects were confirmed previously with a mean difference between jump heights of 1.53 cm after 5 min vs. pre (8). The clinical importance of this result is reinforced by the small ES reported for the comparisons between 9 min post and pre plyometric CA and by the significant difference between conditions (plyometric CA vs. control) reported at 9 min. Furthermore, an increase in CMJ 10 min after a plyometric CA was reported previously with collegiate soccer players (9). Therefore, further data analysis, not only based on the *P*-value, led us to accept the hypothesis raised that plyometric CA increased CMJ performance for up to 9 min after this CA. Other studies are suggested to confirm this interpretation.

A limitation of the present study is the non-probabilistic sampling by volunteering used in the present study. Furthermore, the sample included both males and females, which may have contributed to the large standard deviation shown, which could have influenced the lack of a significant difference at 9 min, for example. Sample stratification and equalization by sex could reduce this limitation. However, due to the difficulty of using probabilistic sampling, we decided to assume this limitation and conduct the study through non-probabilistic sampling and allowing the inclusion of participants of both sexes. Despite this limitation, the included sample was sufficient to demonstrate significant differences for time, condition, and interaction, and the results found are in line with previous reports (3, 8). Another point to highlight was the permanence of participants sitting in the control condition. This was proposed with the aim of submitting participants to a rest period. It is possible that remaining standing, a condition closer to the experimental procedure, would reduce the chance of any intervening variables. However, the close results found 1 min post-control compared to pre suggest that remaining seated did not interfere in the results found. Future studies should be performed aiming to reduce the reported limitations and reinvestigate PAPE responses after a plyometric CA.

The present study demonstrates that performing multiple sets of a plyometric CA improved subsequent performance in the CMJ. It is possible to conclude that multiple sets of a plyometric CA promote PAPE effects up to 9-min after the CA. The results found corroborate with the idea of an earlier PAPE effect promoted by a plyometric CA. Therefore, strength and conditioning coaches and practitioners may consider performing multiple sets of plyometric CA, especially using the protocol reported in the present study (2 × 5 rep with 1 min of rest between, of CMJ, scissor jump, and horizontal jump = totalizing 30 jumps) to produce immediate enhancement of power in the lower limbs.

## Data Availability

The raw data supporting the conclusions of this article will be made available by the authors, without undue reservation.
